# A comparison of pediatric inflammatory multisystem syndrome temporarily-associated with SARS-CoV-2 and Kawasaki disease

**DOI:** 10.1038/s41598-022-26832-5

**Published:** 2023-01-20

**Authors:** Markus Hufnagel, Jakob Armann, André Jakob, Maren Doenhardt, Natalie Diffloth, Anton Hospach, Dominik T. Schneider, Andreas Trotter, Martin Roessler, Jochen Schmitt, Reinhard Berner, The PIMS-DGPI Working Group, Grazyna Adamiak-Brych, Martina Aderhold, Sara Aggar, Mohammed-Ahmed Ahmed, Sandra Akanbi, Kristin Anders, Stefan Arens, Jakob Armann, Christoph Baßmann, Lisa Baumbach, Otto-Jonas Bayrhof, Gerald Beier, Ardua Berger, Daniel Bernard, Reinhard Berner, Mario Berwald, Adina Biering, Ulrike Blümlein, Stefanie Blume, Kai Böckenholt, Carsten Bölke, Thomas Boesing, Robert Bonacker, Monika-Maria Borchers, Britta Brenner, Folke Brinkmann, Jasmin Brühler, Jürgen Brunner, Laura Buchtala, Jörg Budde, Reinhard Bullmann, Marc Carré Schoppe, Gordana Cvetanovic, Alina Czwienzek, Metin Degirmenci, Fenja Dejas, Bergüzar Demirdelen, Anke Diederichs, Maren Dittrich, Katharina Döhring, Helena Donath, Franziska Ebert, Annemarie Eff, Kerstin Ehrentraut, Fiene Eißler, Anne Eißner, Elisa Endres, Matthias Engler, Andreas Fiedler, Karin Fingerhut, Agnes Finster, Doris Fischer, Simon Flümann, Svenja Foth, Christian Fremery, Holger Frenzke, Lukas Galow, Monika Gappa, Stephan Gerling, Stina Gitzinger, Nicola Glaser, Karoline Goj, Sarah Christina Goretzki, Katrin Gröger, Tim Groteclaes, Judith Grüner, Mike Grünwedel, Stephan Haag, Lisa Hacker, Nikolaus Halwas, Christof Hanke, Anne Haupt, Christina Heinrich, Julia Heinrich, Lutz Hempel, Matthias Hermann, Matthias Herzog, Georg Heubner, Georg Hillebrand, Matthias Himpel, Kai-Alexandra Hilker, Cara Hittmeyer, Alexander Höche, Mirjam Höfgen, Uwe Höpner, Katharina Holtkamp, Thomas Hoppen, Marita Horstkemper, Judith Horstmann, Anton Hospach, Markus Hufnagel, Nora Ido, Vladimir Iliaev, Phryne Ioannou, André Jakob, Dirk Jantzen, Söhnke Jenssen, Claudia Jung, Petra Kaiser-Labusch, Herrmann Kalhoff, Johanna Keck, Felicitas Kelch, Thomas Keller, Svetlana Kelzon, Jan Kern, Marie-Sophie Keßner, Daniel Kever, Arni Kirchner, Martin Kirschstein, Richard Kitz, Dietrich Klauwer, Christine Kleff, Christof Kluthe, Jan Knechtel, Lisanne Knop, Holger Köster, Malte Kohns Vasconcelos, Florian Konrad, Robert Kosteczka, Georgia Koukli, Sascha Kowski, Mirco Kuhnigk, Marion Kuska, Sachicko Kwaschnowitz, Veit Lange, Gerrit Lautner, Hanna Libuschewski, Johannes Liese, Linus Lindemann, Norbert Lorenz, Niko Lorenzen, Daniela Lubitz, Heike Machata, Franziska Mader, Ingrid Malath, Cornelie Mampe, Andrea Markowsky, Maximilian Mauritz, Jochen Meister, Melanie Menden, Felix Menzel, Michael Merker, Jens Meyer, Lars Meyer-Dobkowitz, Marko Mohorovicic, Laura Gabriela Moise, Yvonne Morawski, Laura Motzkus, Bianca Müller, Guido Müller, Mirja Müller, Meike Müller, Thomas Niehaus, Andre Oberthür, Johanna Ohlendorf, Florina Olar, Bernd Opgen-Rhein, Iris Östreicher, Kathlee Parthey, Falk Pentek, Simone Pötzsch, Corinna Ponsa, Jenny Rambow, Heike Reck, Friedrich Reichert, Annika Reil, Thomas Reinhardt, Carolin Richter, Jost Wigand Richter, Nikolaus Rieber, Hannelore Ringe, Alexander Rühlmann, Anja Samol, Kristin Sauerbrey, Miriam Schäfer, Nico Schaeffer, Miriam Scheffler, Christian Schlick, Caroline Schmitt, Dominik Schneider, Hans-Christoph Schneider, Alexander Schnelke, Roland Schrewe, Lothar Schrod, Oliver Schroers, Katharina Schütz, Leonie Schulteß, Isabel Schumacher, Sabrina Seidel, Arne Simon, Volker Soditt, Ezgi Sönmez, Elena Spancken, Lisa Spath, Sebastian Spinner, Barbara Stastny, Michael Steidl, Benedikt Steif, Ann-Kathrin Steimer, Frank Stemberg, Antje Stockmann, Thomas Stöhring, Daria Sumbadze, Axel Teichmann, Lion Thiel, Jan Tränkner, Stefanie Trau, Tina Treichel, Andreas Trotter, Alijda van den Heuvel, Kai Vehse, Lena Vischer, Tatjana Wahjudi, Karin Waldecker, Ulrike Walden, Laura Warneke, Sarah Weber, Götz Wehl, Falk Wehrhoff, Alexander Weigert, Sandra Wenzel, Annika Werner, Clarissa Weste, Barbara Wichmann, Florian Wild, Denise Willmer, Felicitas Wolf, Nina Wrenger, Donald Wurm, Anne-Sophie Yussif, Yvonne Zeißig, Ulrich Zügge

**Affiliations:** 1 Division of Pediatric Infectious Diseases and Rheumatology, Department of Pediatrics and Adolescent Medicine, University Medical Center, Medical Faculty, University of Freiburg, Mathildenstr. 1, 79106 Freiburg, Germany; 2 Department of Pediatrics, University Hospital, Medical Faculty Carl Gustav Carus, Technische Universität Dresden, Dresden, Germany; 3 Department of Pediatric Cardiology and Pediatric Intensive Care, Ludwig Maximilians University of Munich, Munich, Germany; 4 Olga-Hospital, Stuttgart, Germany; 5 Clinic of Pediatrics, Municipal Hospital Dortmund, Dortmund, Germany; 6 Children’s Hospital and Center for Perinatal Medicine, Singen, Germany; 7 Center for Evidence-Based Healthcare, Medical Faculty Carl Gustav Carus, Technische Universität Dresden, Dresden, Germany; 8 Klinik für Kinder- und Jugendmedizin, Rodewisch, Germany; 9 Klinik für Kinder- und Jugendmedizin, Lüneburg, Germany; 10 Klinik für Kinder- und Jugendmedizin, Karlsruhe, Germany; 11 Klinik für Kinder- und Jugendmedizin, Schwelm, Germany; 12 Klinik für Kinder- und Jugendmedizin Berlin-Neukölln, Berlin, Germany; 13 Klinik für Kinder- und Jugendmedizin Leipzig-St. Georg, Leipzig, Germany; 14 Kinder- und Jugendkrankenhaus, Hannover, Germany; 15 Kinderklinik, Darmstadt, Germany; 16 Klinik für Kinder- und Jugendmedizin Universitätsklinikum, Ulm, Germany; 17 Klinik für Kinder- und Jugendliche, Schweinfurt, Germany; 18 Klinik für Kinder- und Jugendmedizin, Deggendorf, Germany; 19 Klinik für Kinder- und Jugendliche, Esslingen, Germany; 20 Klinik für Kinderheilkunde Universitätsklinikum Essen, Essen, Germany; 21 Klinik für Kinder- und Jugendmedizin, Rüsselsheim, Germany; 22 Klinik für Kinder- und Jugendmedizin, Wolfsburg, Germany; 23 Klinik für Kinder- und Jugendmedizin, Cottbus, Germany; 24 Klinik für Kinder- und Jugendmedizin, Detmold, Germany; 25 Klinik für Kinder- und Jugendmedizin, Köln, Germany; 26 Klinik für Kinder- und Jugendliche, Ravensburg, Germany; 27 Klinik für Kinder- und Jugendmedizin, Bielefeld, Germany; 28 Klinik für Kinder- und Jugendmedizin Universitätsklinikum, Lübeck, Germany; 29 Klinik für Kinder- und Jugendmedizin München-Nymphenburg, München, Germany; 30 Klinik für Kinder- und Jugendmedizin Universitätsklinikum, Bochum, Germany; 31 Klinik für Kinder- und Jugendmedizin, Freudenstadt, Germany; 32 Klinik für Kinder- und Jugendheilkunde Universitätsklinikum, Innsbruck, Austria; 33 Prof.-Hess Kinderklinik Bremen-Mitte, Bremen, Germany; 34 Klinik für Kinder- und Jugendmedizin RKK-Klinikum Freiburg, Freiburg im Breisgau, Germany; 35 Klinik für Kinder- und Jugendmedizin, Bad Kreuznach, Germany; 36 Klinik für Kinder- und Jugendmedizin, Ludwigshafen, Germany; 37 Klinik für Kinder- und Jugendmedizin, Johannes Wesling Klinikum Minden, Minden, Germany; 38 Haunersche Kinderklinik, München, Germany; 39 Kinderklinik, Duisburg, Germany; 40 Klinik für Kinder- und Jugendmedizin, Pforzheim, Germany; 41 Kinderzentrum, Hildesheim, Germany; 42 Kinderkrankenhaus Hamburg-Altona, Hamburg, Germany; 43 Klinik für Kinder- und Jugendmedizin, Dinslaken, Germany; 44 Klinik für Kinder- und Jugendmedizin Universitätsklinikum, Frankfurt, Germany; 45 Klinik für Kinder- und Jugendmedizin, Lichtenstein, Germany; 46 Klinik für Kinder- und Jugendmedizin, Altenburger Land, Germany; 47 Klinik für Kinder- und Jugendmedizin Berlin-Lichtenberg, Berlin, Germany; 48 Kinderkrankenhaus Hamburg-Wilhemstift, Hamburg, Germany; 49 Klinik für Kinder- und Jugendmedizin, Offenbach, Germany; 50 Klinik für Kinder- und Jugendliche St. Marien, Amberg, Germany; 51 Klinik für Kinder- und Jugendmedizin, Limburg, Germany; 52 Zentrum für Kinder- und Jugendliche, Wesel, Germany; 53 Klinik für Kinder- und Jugendmedizin Universitätsklinikum, Marburg, Germany; 54 Klinik für Kinder- und Jugendmedizin, Wiesbaden, Germany; 55 Klinik für Kinder- und Jugendliche, Lüdenscheid, Germany; 56 Klinik für Kinder- und Jugendliche, Düsseldorf, Germany; 57 Klinik und Poliklinik für Kinder- und Jugendmedizin Universitätsklinikum Regensburg, Regensburg, Germany; 58 Klinik für Kinder- und Jugendmedizin, Trier, Germany; 59 Klinik für Kinder- und Jugendmedizin Berlin-Westend, Berlin, Germany; 60 Klinik für Kinder- und Jugendmedizin, Suhl, Germany; 61 Klinik für Kinder- und Jugendmedizin, Wurzen, Germany; 62 Klinik für Kinderheilkunde und Jugendmedizin und Kinderchirurgie, Villingen-Schwenningen, Germany; 63 Klinik für Kinder- und Jugendliche, Bamberg, Germany; 64 Klinik für Kinder- und Jugendmedizin, Neuburg, Germany; 65 Klinik für Kinder- und Jugendliche, Schwäbisch Hall, Germany; 66 Klinik für Kinder- und Jugendmedizin, Zwickau, Germany; 67 Klinik für Kinder- und Jugendmedizin, Speyer, Germany; 68 Waldklinikum Gera, Gera, Germany; 69 Klinik für Kinder- und Jugendmedizin Aschaffenburg-Alzenau, Aschaffenburg, Germany; 70 Klinik für Kinder- und Jugendmedizin Dresden-Neustadt, Dresden, Germany; 71 Klinik für Kinder- und Jugendmedizin Itzehoe, Itzehoe, Germany; 72 Klinikum Eichsfeld, Eichsfeld, Germany; 73 Kinderklinik, Hagen, Germany; 74 Kinderklinik, Gummersbach, Germany; 75 Klinik für Kinder- und Jugendheilkunde, Landau, Germany; 76 Klinik für Kinder- und Jugendmedizin, Chemnitz, Germany; 77 Klinik für Kinder- und Jugendmedizin, Koblenz, Germany; 78 Kinderklinik Köln-Porz, Köln, Germany; 79 Klinik für Kinder- und Jugendmedizin, Stollberg, Germany; 80 Klinik für Kinder- und Jugendmedizin, Bad Mergentheim, Germany; 81 Klinik für Kinder- und Jugendmedizin, Eberswalde, Germany; 82 Klinik für Kinder- und Jugendmedizin, Saarbrücken, Germany; 83 Klinik für Kinder- und Jugendmedizin Berlin-Buch, Berlin, Germany; 84 Klinik für Kinder- und Jugendliche Josefinum, Augsburg, Germany; 85 Klinik für Kinder- und Jugendmedizin, Sankt Augustin, Germany; 86 Universitätsklinik für Kinder- und Jugendmedizin Tübingen, Tübingen, Germany; 87 Klinik für Kinder- und Jugendliche, Mönchengladbach, Germany; 88 Klinik für Kinder- und Jugendmedizin, Rosenheim, Germany; 89 Klinik für Kinder- und Jugendmedizin, Celle, Germany; 90 Kinderhospital, Frankfurt, Germany; 91 Klinik für Kinder- und Jugendliche, Rothenburg ob der Tauber, Germany; 92 Kinder- und Jugendklinik, Witten, Germany; 93 Klinik für Kinder- und Jugendmedizin St. Franziskus-Hospital, Münster, Germany; 94 Zentrum für Kinder- und Jugendmedizin, Oldenburg, Germany; 95 Klinik für Kinder- und Jugendmedizin Universitätsklinikum Düsseldorf, Düsseldorf, Germany; 96 Klinik für Kinderheilkunde und Jugendmedizin, Offenburg, Germany; 97 Klinik für Kinder- und Jugendmedizin, Kaiserslautern, Germany; 98 Klinik für Kinder- und Jugendmedizin, Bottrop, Germany; 99 Klinik für Kinder- und Jugendmedizin Augsburg, Augsburg, Germany; 100 Klinik für Kinder- und Jugendmedizin, Winnenden, Germany; 101 Kinder- und Jugendklinik Gelsenkirchen, Gelsenkirchen, Germany; 102 Kinder- und Poliklinik Universitätsklinikum Würzburg, Würzburg, Germany; 103 Klinik für Kinder- und Jugendmedizin, Kiel, Germany; 104 Klinik für Kinderheilkunde, Düsseldorf, Germany; 105 Klinik für Kinder- und Jugendmedizin Berlin-Spandau, Berlin, Germany; 106 Klinik für Kinder- und Jugendmedizin, Herford, Germany; 107 Klinik für Kinder- und Jugendliche, Neuss, Germany; 108 Klinik für Kinder- und Jugendmedizin, Freiberg, Germany; 109 Kinder- und Jugendklinik, Datteln, Germany; 110 Klinik für Kinder- und Jugendmedizin, Aue, Germany; 111 Zentrum für Kinder- und Jugendmedizin, Osnabrück, Germany; 112 Klinik für Kinder- und Jugendmedizin Berlin-Tempelhof, Berlin, Germany; 113 Klinik für Kinder- und Jugendmedizin, Neuwied, Germany; 114 Kinder- und Jugendklinik, Potsdam, Germany; 115 Klinik für Kinder- und Jugendmedizin, Erfurt, Germany; 116 Kinder- und Jugendmedizin Uniklinik Köln, Köln, Germany; 117 Klinik für Pädiatrie Universitätsklinikum Hannover, Hanover, Germany; 118 Klinik für Kinder- und Jugendmedizin, Wittlich, Germany; 119 Klinik für Pädiatrie, Charité Universitätsmedizin Berlin, Berlin, Germany; 120 Pädiatrie Universitätsklinikum Halle, Halle, Germany; 121 Klinik für Kinder- und Jugendmedizin, Plauen, Germany; 122 Klinik für Kinder- und Jugendmedizin, Zittau, Germany; 123 Klinik für Kinder- und Jugendmedizin, Krefeld, Germany; 124 Klinik für Kinder- und Jugendmedizin Universitätsklinikum Jena, Jena, Germany; 125 Klinik für Kinder- und Jugendmedizin, Braunschweig, Germany; 126 Klinik und Poliklinik für Kinder- und Jugendmedizin Universitätsklinikum Müchen-Schwabing, München, Germany; 127 Klinik für Kinder- und Jugendmedizin Chemnitz-Rabenstein, Chemnitz, Germany; 128 Klinikum Freising, Freising, Germany; 129 Cnopf’sche Kinderklinik, Nürnberg, Germany; 130 Kinder- und Jugendmedizin Mariahilf-Klinik, Hamburg, Germany; 131 Klinik für Kinder- und Jugendmedizin, Reutlingen, Germany; 132 Klinik für Kinder- und Jugendmedizin, Landsberg am Lech, Germany; 133 Klinik für Kinder- und Jugendmedizin Frankfurt-Höchst, Frankfurt, Germany; 134 Fachabteilung für Kinder- und Jugendmedizin, Garmisch-Partenkirchen, Germany; 135 Klinik für Allgemeine Pädiatrie und Neonatologie Universitätsklinikum Homburg, Homburg, Germany; 136 Klinik für Kinder- und Jugendliche, Solingen, Germany; 137 Klinik für Kinder- und Jugendmedizin, Kassel, Germany; 138 Kinderklinik, Passau, Germany; 139 Klinik für Kinder- und Jugendmedizin, Worms, Germany; 140 Klinik für Kinder- und Jugendliche, Oberhausen, Germany; 141 Klinik für Kinderheilkunde und Jugendmedizin, Neustadt am Rübenberge, Germany; 142 Klinik für Kinder- und Jugendmedizin Universitätsklinikum Münster, Münster, Germany; 143 Klinik für Kinder- und Jugendmedizin, Stade, Germany; 144 Klinik für Kinder- und Jugendmedizin, Konstanz, Germany; 145 Klinik für Kinder- und Jugendmedizin, Böblingen, Germany; 146 Pädiatrie Klinikum Kaufbeuren, Kaufbeuren, Germany; 147 Zentrum für Kinderheilkunde Universitätsklinikum Bonn, Bonn, Germany; 148 Klinik für Kinder- und Jugendmedizin, Salzgitter, Germany; 149 Kinder- und Jugendklinik Universitätsklinikum Rostock, Rostock, Germany; 150 Kinder- und Jugendklinik, Coesfeld, Germany; 151 Klinik für Kinder- und Jugendmedizin, Velbert, Germany

**Keywords:** Epidemiology, Viral infection

## Abstract

The connection between Pediatric Inflammatory Multisystem Syndrome (PIMS) and Kawasaki Disease (KD) is not yet fully understood. Using the same national registry, clinical features and outcome of children hospitalized in Germany, and Innsbruck (Austria) were compared. Reported to the registry were 395 PIMS and 69 KD hospitalized patients. Patient age in PIMS cases was higher than in KD cases (median 7 [IQR 4–11] vs. 3 [IQR 1–4] years). A majority of both PIMS and KD patients were male and without comorbidities. PIMS patients more frequently presented with organ dysfunction, with the gastrointestinal (80%), cardiovascular (74%), and respiratory (52%) systems being most commonly affected. By contrast, KD patients more often displayed dermatological (99% vs. 68%) and mucosal changes (94% vs. 64%), plus cervical lymph node swelling (51% vs. 34%). Intensive care admission (48% vs. 19%), pulmonary support (32% vs. 10%), and use of inotropes/vasodilators (28% vs. 3%) were higher among PIMS cases. No patients died. Upon patient discharge, potentially irreversible sequelae—mainly cardiovascular—were reported (7% PIMS vs. 12% KD). Despite differences in age distribution and disease severity, PIMS and KD cases shared many common clinical and prognostic characteristics. This supports the hypothesis that the two entities represent a syndrome continuum.

## Introduction

With its start in December 2019, Severe Acute Respiratory Syndrome Coronavirus-2 (SARS-CoV-2) has emerged as a global pandemic^1^. In contrast to its course in adults, among children and adolescents, Coronavirus Disease-19 (COVID-19) usually is mild and has a low hospitalization rate^2–4^. In April 2020, a multisystem inflammatory syndrome associated with SARS-CoV-2 first was observed among children in Europe and North America. The World Health Organization (WHO) named this syndrome Pediatric Multisystem Inflammatory Syndrome Temporarily associated with SARS-CoV-2 (PIMS-TS)^5^. Multisystem Inflammatory Syndrome in Children (MIS-C) is a synonymous term proposed by the Centers of Disease Control^6^.

PIMS, the term we have adopted for use with our survey, is a clinical, heterogenous syndrome that partially overlaps with both Kawasaki Disease (KD) and Toxic Shock Syndrome^7–9^. Most comparisons between PIMS and KD have been drawn from historical, rather than from concurrent, cohorts^10,11^. As a result, it has been difficult to determine whether PIMS and KD are different diseases or whether they instead may be part and parcel of the same syndrome—with the two on a spectrum ranging from less severe (KD) to more severe (PIMS)^12,13^. To address this question, the ability to investigate the emergence of PIMS and KD cases simultaneously and in parallel is critical.

Beginning on March 18, 2020, the German Society for Pediatric Infectious Diseases (DGPI), with the support of several other German professional pediatric societies, has collected nationwide data on children and adolescents hospitalized with PIMS in Germany, as well as in the neighboring city of Innsbruck, Austria. As a comparison group, Kawasaki disease (KD) cases *not* associated with SARS-CoV-2 were collected through the same survey. With a combined dataset of 395 PIMS and 69 KD cases, we compared detailed information on clinical characteristics, disease course and outcome parameters.

## Results

### Study population and demographics

Between March 18, 2020 and August, 31, 2021, 154 institutions reported 517 patients to the registry. The first recorded admission was on January 7, 2020. Fifty-three patients (10%) were excluded from the analysis, either because they did not meet the PIMS criteria, or because their dataset was incomplete (Supplementary Table 1). The final dataset included 464 patients. Of these, 395 patients were classified as PIMS (PIMS-all, 85%), 242 patients as PIMS with Kawasaki disease features (PIMS-KD, 52%), 153 patients as PIMS without Kawasaki disease features (PIMS-non-KD, 33%), and 69 as Kawasaki disease without association with SARS-CoV-2 infection (KD, 15%). The ratio of PIMS-all as compared to KD was 5.7:1. In comparison to PIMS-KD, more KD cases were complete (54% [37/69] vs. 33% [79/242]).

**Figure 1:**
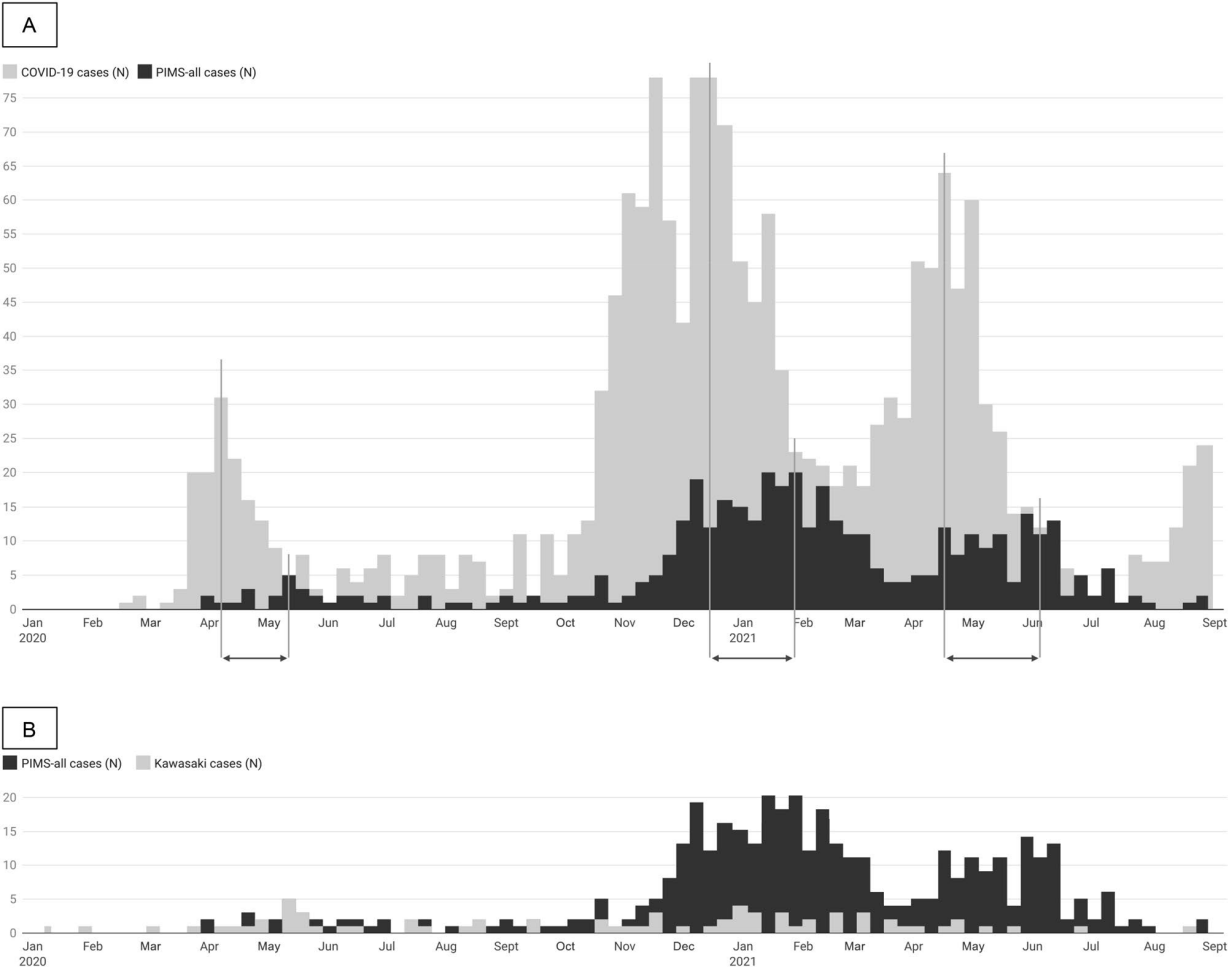
Weekly numbers of hospital-admitted cases of (**A**) pediatric COVID-19 and PIMS-all, as well as of cases with (**B**) PIMS-all and KD. Graphics created by using the software from www.datawrapper.de.

The number of PIMS-all patients hospitalized per week (between 0 and 20 cases) peaked at three timepoints during our study period: first in May 2020, then from December 2020 to February 2021, and then again in May–June 2021 (Fig. 1A). PIMS-all cases began to rise 5–7 weeks following a spike of COVID-19 hospitalizations (Fig. 1A). When the number of SARS-CoV-2 infections went down, PIMS-all cases also decreased 5–7 weeks later. By contrast, KD cases were more evenly distributed (0–5 cases per week over the 18-month period; Fig. 1B). Interestingly, however, after May 2021, the number of KD cases reported decreased.

**Figure 2:**
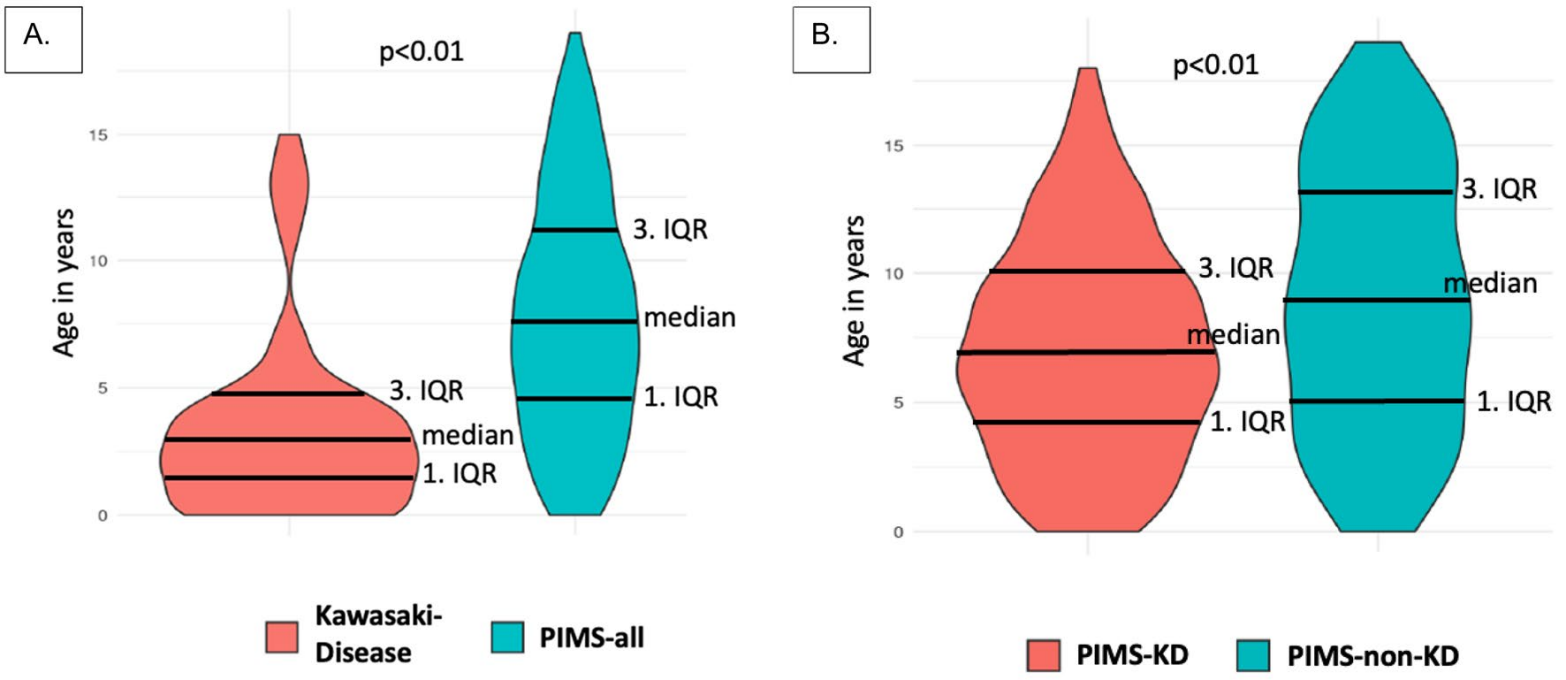
Age distribution of patients with (**A**) PIMS-all and KD, as well as of patients with (**B**) PIMS-non-KD and PIMS-KD.

An important difference between PIMS-all and KD cases emerged in connection with patient age. In comparison with KD, PIMS-all patients were significantly older (Table 1, Fig. 2). While the median age of PIMS-all was 7 years, as compared to 3 years in KD cases, PIMS-KD were younger than PIMS-non-KD patients (Fig. 2). Incidence by age group in PIMS patients was highest among 7- to 15-year-old children. Male patients more commonly were affected by both PIMS and KD than were female patients (1.8:1 ratio in PIMS vs. 1.7:1 ratio in KD; Table 1). In addition, it was more common (*p* = 0.025) for KD patients to be Caucasian than it was for PIMS-all patients (Table 1). By contrast, no difference in ethnicity distribution was observed when comparing PIMS-non-KD and PIMS-KD cases. Preexisting comorbidities, most often respiratory and/or cardiovascular-related, were present in 21–29% of both KD and PIMS cases (Table 1).

**Table 1:** Clinical characteristics of patients with PIMS-all, PIMS-non-KD, PIMS-KD and KD.

	PIMS-all (n = 395)	PIMS-non-KD (n = 153)	PIMS-KD (n = 242)	*p*-value^a^	KD	*p*-value^b^
				(95% CI)	(n = 69)	(95% CI)
**Age**, median in years (IQR)	7 (4–11)	9 (5–13)	7 (4–10)	** < 0.001** (n.d.)	3 (1–4)	** < 0.001** (n.d.)
< 1 y, n (%)	13 (3.3)	5 (3.3)	8 (3.3)		13 (18.8)	
1–6 y, n (%)	156 (39.5)	47 (30.7)	109 (45.0)		45 (65.2)	
7–15 y, n (%)	199 (50.4)	80 (52.3)	119 (49.2)		11 (15.9)	
16–19 y, n (%)	27 (6.8)	21 (13.7)	6 (2.5)		0	
**Sex**						
Female, n (%)	141 (35.7)	55 (35.9)	86 (35.5)	1.000 (n.d.)	26 (37.7)	0.786 (n.d.)
Male, n (%)	254 (64.3)	98 (64.1)	156 (64.5)		43 (62.3)	
Male-to-female ratio	1:08	1:08	1:08		1:07	
**Ethnicity**						
Caucasian, n (%)	226 (57.2)	89 (58.2)	137 (56.6)	0.631 (n.d.)	47 (68.1)	**0.025** (n.d.)
African, n (%)	26 (6.6)	9 (5.9)	17 (7.0)		2 (2.9)	
Arabic, n (%)	64 (16.2)	25 (16.3)	39 (16.1)		6 (8.7)	
Asian, n (%)	18 (4.6)	4 (2.6)	14 (5.8)		8 (11.6)	
Other, n (%)	61 (15.4)	26 (17.0)	35 (14.5)		6 (8.7)	
**Concomitant diseases**	94 (23.8)	44 (28.8)	50 (20.7)	0.430 (− 10.2 to 4.4)	15 (21.7)	0.440 (− 13.0 to 5.7)
Respiratory, n (%)	18 (4.6)	7 (4.6)	11 (4.5)	0.990 (− 4.3 to 4.2)	1 (1.4)	0.230 (− 2.0 to 8.2)
Cardiovascular, n (%)	13 (3.3)	4 (2.6)	9 (3.7)	0.550 (− 2.5 to 4.7)	3 (4.3)	0.660 (− 5.7 to 3.6)
Gastrointestinal, n (%)	9 (2.3)	4 (2.6)	5 (2.1)	0.720 (− 3.6 to 2.5)	0	0.210 (− 1.3 to 5.8)
Hepatic, n (%)	2 (0.5)	2 (1.3)	0	0.070 (− 2.7 to 0.1)	0	0.550 (− 1.2 to 2.2)
Renal, n (%)	4 (1.0)	2 (1.3)	2 (0.8)	0.640 (− 2.5 to 1.6)	2 (2.9)	0.200 (− 4.8 to 1.0)
Neurological, n (%)	7 (1.8)	4 (2.6)	3 (1.2)	0.310 (− 4.1 to 1.3)	1 (1.4)	0.850 (− 3.0 to 3.7)
Psychiatric, n (%)	4 (1.0)	2 (1.3)	2 (0.8)	0.640 (− 2.5 to 1.6)	0	0.400 (− 1.4 to 3.4)
Hematological, n (%)	8 (2.0)	4 (2.6)	4 (1.7)	0.510 (− 3.8 to 1.9)	1 (1.4)	0.750 (− 3.0 to 4.1)
Oncological, n (%)	1 (0.3)	0	1 (0.4)	0.430 (− 0.6 to 1.4)	0	0.680 (− 0.9 to 1.4)
Organ/Bone marrow transplant, n (%)	1 (0.3)	1 (0.7)	0	0.210 (− 1.7 to 0.4)	0	0.680 (− 0.9 to 1.4)
Autoimmune, n (%)	8 (2.0)	4 (2.6)	4 (1.7)	0.510 (− 3.8 to 1.9)	3 (4.3)	0.240 (− 6.2 to 1.6)
Immunodeficiency, n (%)	1 (0.3)	1 (0.7)	0	0.210 (− 1.7 to 0.4)	0	0.680 (− 0.9 to 1.4)
Immunosuppressive drug, n (%)	2 (0.5)	1 (0.7)	1 (0.4)	0.740 (− 1.7 to 1.2)	0	0.550 (− 1.2 to 2.2)
Others, n (%)	16 (4.1)	8 (5.2)	8 (3.3)	0.350 (− 5.9 to 2.1)	4 (5.8)	0.510 (− 7.0 to 3.5)

PIMS, pediatric multisystem inflammatory syndrome; KD, Kawasaki disease. ^a^comparison between PIMS-non-KD and PIMS-KD. ^b^comparison between PIMS-all and KD; 95% CI, 95%-confidence interval; n; number of cases; IQR, interquartile range; y, years; n.d.; not determined. *P* values in bold are considered statistically significant.

### Clinical characteristics

Among PIMS-all patients, the most common organ involvements were gastrointestinal (80%), cardiovascular (74%), dermatological (68%) and mucosal membranes (64%) (Table 2). Compared to KD, PIMS-all patients more commonly presented with symptoms relating to the gastrointestinal (including ascites), cardiovascular, respiratory, hematological, neurological and renal organ systems. By contrast, KD patients more commonly presented with dermatological, mucosal membrane (including conjunctivitis) and ear-nose-throat symptoms, along with cervical lymphadenopathy, arthralgias and arthritis. Unlike PIMS-non-KD, PIMS-KD patients more commonly presented with dermatological, mucosal membrane (including conjunctivitis) and ear-nose-throat symptoms, along with cervical lymphadenopathy, vomiting, hepatosplenomegaly and anemia. PIMS-non-KD patients more commonly had tachy-/dyspnea, pneumonia, pediatric acute respiratory distress syndrome (pARDS), thickening of enteric walls, and nephritis. Between PIMS-all and PIMS-KD patients, there were no differences in cardiovascular presentation.

**Table 2:** Clinical symptoms and significant laboratory values in patients with PIMS-all, PIMS-non-KD, PIMS-KD and KD.

	PIMS-all (n = 395)	PIMS-non-KD (n = 153)	PIMS-KD (n = 242)	*p*-value^a^ (95% CI)	KD (n = 69)	*p*-value^b^ (95% CI)
**Ear-nose-throat (ENT)**, n (%)	119 (30.1)	n.sh	n.sh	n.sh	32 (46.4)	**0.010** (− 28.2 to 4.3)
**Respiratory**, n (%)	204 (51.6)	80 (52.3)	124 (51.2)	0.840 (− 11.2 to 9.1)	23 (33.3)	** < 0.001** (5.6–31.0)
Dyspnea, n (%)	44 (11.1)	26 (17.0)	18 (7.4)	** < 0.001** (− 15.9 to 3.2)	4 (5.8)	0.180 (− 2.5 to 13.2)
Tachypnea, n (%)	85 (21.5)	47 (30.7)	38 (15.7)	** < 0.001** (− 23.2 to 6.8)	7 (10.1)	**0.030** (1.2–21.6)
Pleural effusion, n (%)	116 (29.4)	40 (26.1)	76 (31.4)	0.260 (− 4.0 to 14.5)	8 (11.6)	** < 0.001** (6.5–29.0)
Pneumonia, n (%)	63 (15.9)	34 (22.2)	29 (12.0)	**0.010** (− 17.6 to 2.9)	5 (7.2)	0.060 (− 0.3 to 17.8)
Pediatric acute respiratory distress syndrome (pARDS), n (%)	32 (8.1)	21 (13.7)	11 (4.5)	** < 0.001** (− 14.7 to 3.7)	1 (1.4)	0.050 (0.1 to 13.2)
**Cardiovascular**, n (%)	294 (74.4)	108 (70.6)	186 (76.9)	0.160 (− 2.6 to 15.1)	42 (60.9)	**0.020** (2.1–25.0)
Heart failure, n (%)	110 (27.8)	41 (26.8)	69 (28.5)	0.710 (− 7.4 to 10.8)	4 (5.8)	** < 0.001** (11.2–32.9)
Arterial hypotension, n (%)	84 (21.3)	30 (19.6)	54 (22.3)	0.520 (− 5.6 to 11.0)	4 (5.8)	** < 0.001** (5.5–25.4)
Pericardial effusion, n (%)	134 (33.9)	50 (32.7)	84 (34.7)	0.680 (− 7.6 to 11.7)	15 (21.7)	0.050 (0.2–24.1)
Myocardial dysfunction, n (%)	161 (40.8)	62 (40.5)	99 (40.9)	0.940 (− 9.6 to 10.4)	8 (11.6)	** < 0.001** (17.1–41.2)
Peri-/Myocarditis, n (%)	101 (25.6)	41 (26.8)	60 (24.8)	0.660 (− 10.9 to 6.9)	5 (7.2)	** < 0.001** (7.7–29.0)
Coronary artery dilatation, n (%)	44 (11.1)	13 (8.5)	31 (12.8)	0.190 (− 2.1 to 10.7)	18 (26.1)	** < 0.001** (− 23.6 to 6.3)
Coronary aneurysm, n (%)	37 (9.4)	11 (7.2)	26 (10.7)	0.430 (− 2.4 to 9.5)	13 (18.8)	**0.020** (− 17.4 to 1.6)
**Gastrointestinal**, n (%)	316 (80.0)	116 (75.8)	200 (82.6)	0.100 (− 1.3 to 14.9)	40 (58.0)	** < 0.001** (11.4–32.7)
Abdominal pain, n (%)	165 (41.8)	67 (43.8)	98 (40.5)	0.520 (− 13.3 to 6.7)	20 (29.0)	0.050 (0.3–25.3)
Vomiting, n (%)	151 (38.2)	49 (32.0)	102 (42.1)	**0.040** (0.3–20.0)	26 (37.7)	0.930 (− 11.9 to 13.0)
Diarrhea, n (%)	174 (44.1)	61 (39.9)	113 (46.7)	0.180 (− 3.3 to 16.9)	27 (39.1)	0.450 (− 7.8 to 17.6)
Ascites, n (%)	126 (31.9)	44 (28.8)	82 (33.9)	0.290 (− 4.3 to 14.6)	12 (17.4)	**0.010** (2.8–26.2)
Gastroenteritis	75 (19.0)	22 (14.4)	53 (21.9)	0.060 (− 0.4 to 15.5)	9 (13.0)	0.240 (− 3.9 to 15.8)
Appendicitis, n (%)	27 (6.8)	15 (9.8)	12 (5.0)	0.060 (− 10.0 to 0.3)	3 (4.3)	0.440 (− 3.8 to 8.8)
Peritonitis, n (%)	22 (5.6)	11 (7.2)	11 (4.5)	0.270 (− 7.3 to 2.0)	1 (1.4)	0.150 (− 1.4 to 9.7)
Thickening of enteric wall, n (%)	76 (19.2)	40 (26.1)	36 (14.9)	**0.010** (− 19.2 to 3.3)	2 (2.9)	** < 0.001** (6.8–25.8)
Splenomegaly, n (%)	68 (17.2)	19 (12.4)	49 (20.2)	**0.040** (0.2–15.5)	9 (13.0)	0.390 (− 5.4 to 13.7)
**Hepatic**, (%)	82 (20.8)	26 (17.0)	56 (23.1)	0.140 (− 2.1 to 14.4)	13 (18.8)	0.720 (− 8.4 to 12.3)
Hepatomegaly, n (%)	64 (16.2)	15 (9.8)	49 (20.2)	**0.010** (3.0–17.9)	11 (15.9)	0.960 (− 9.2 to 9.7)
**Renal**, n (%)	82 (20.8)	34 (22.2)	48 (19.8)	0.570 (− 10.6 to 5.9)	7 (10.1)	**0.040** (0.5–20.7)
Impaired renal function, n (%)	48 (12.2)	21 (13.7)	27 (11.2)	0.450 (− 9.2 to 4.1)	2 (2.9)	**0.020** (1.3–17.2)
Nephritis, n (%)	5 (1.3)	5 (3.3)	0	** < 0.001** (− 5.5 to 1.0)	2 (2.9)	0.310 (− 4.8 to 1.5)
**Neurological**, n (%)	90 (22.8)	35 (22.9)	55 (22.7)	0.970 (− 8.7 to 8.4)	8 (11.6)	**0.040** (0.8–21.6)
Headache, n (%)	43 (10.9)	16 (10.5)	27 (11.2)	0.830 (− 5.6 to 7.0)	4 (5.8)	0.800 (− 2.6 to 12.8)
Meningitis, n (%)	17 (4.3)	7 (4.6)	10 (4.1)	0.830 (− 4.6 to 3.7)	1 (1.4)	0.260 (− 2.1 to 7.8)
**Psychiatric**, n (%)	15 (3.8)	5 (3.3)	10 (4.1)	0.660 (− 3.0 to 4.8)	1 (1.4)	0.330 (− 2.3 to 7.0)
**Musculoskeletal**, n (%)	40 (10.1)	14 (9.2)	26 (10.7)	0.610 (− 4.5 to 7.7)	10 (14.5)	0.280 (− 12.3 to 3.6)
Arthralgia, n (%)	17 (4.3)	7 (4.6)	10 (4.1)	0.830 (− 4.6 to 3.7)	7 (10.1)	**0.040** (− 11.5 to 0.2)
Arthritis, n (%)	7 (1.8)	3 (2.0)	4 (1.7)	0.820 (− 3.0 to 2.4)	4 (5.8)	**0.040** (− 7.9 to 0.1)
Myalgia, n (%)	27 (6.8)	8 (5.2)	19 (7.9)	0.320 (− 2.5 to 7.8)	3 (4.3)	0.440 (− 3.8 to 8.8)
Myositis, n (%)	4 (1.0)	2 (1.3)	2 (0.8)	0.640 (− 2.5 to 1.6)	0	0.400 (− 1.4 to 3.4)
**Hematological**, n (%)	152 (38.5)	54 (35.3)	98 (40.5)	0.300 (− 4.7 to 15.1)	15 (21.7)	**0.010** (4.5–29.0)
Anemia, n (%)	80 (20.3)	21 (13.7)	59 (24.4)	**0.010** (2.5–18.8)	9 (13.0)	0.160 (− 2.9 to 17.3)
Thrombocytopenia, n (%)	62 (15.7)	18 (11.8)	44 (18.2)	0.090 (− 1.0 to 13.8)	3 (4.3)	**0.010** (2.5–20.2)
Disseminated intravascular coagulation, n (%)	39 (9.9)	18 (11.8)	21 (8.7)	0.320 (− 9.2 to 3.0)	0	**0.010** (2.8–16.9)
**Cutaneous**, n (%)	269 (68.1)	n.sh	n.sh	n.sh	68 (98.6)	** < 0.001** (− 41.6 to 19.3)
Rash, n (%)	229 (58.0)	n.sh	n.sh	n.sh	59 (85.5)	** < 0.001** (− 29.9 to 12.6)
Desquamation, n (%)	35 (8.9)	n.sh	n.sh	n.sh	17 (24.6)	** < 0.001** (− 23.8 to 7.8)
Swelling of hand/feet, n (%)	117 (29.6)	n.sh	n.sh	n.sh	37 (53.6)	** < 0.001** (− 35.9 to 12.1)
**Mucosal**, n (%)	254 (64.3)	n.sh	n.sh	n.sh	65 (94.2)	** < 0.001** (− 41.5 to 18.3)
Enanthema, n (%)	151 (38.2)	n.sh	n.sh	n.sh	47 (68.1)	** < 0.001** (− 42.3 to 17.5)
Conjunctivitis, n (%)	226 (57.2)	n.sh	n.sh	n.sh	60 (87.0)	** < 0.001** (− 41.9 to 17.5)
Cervical lymphadenopathy, n (%)	136 (34.4)	n.sh	n.sh	n.sh	35 (50.7)	**0.010** (− 28.6 to 4.0)
**Hemoglobin**, median in mmol/L, (IQR)	6.2 (5.6–7.2)	6.3 (5.6–7.3)	6.2 (5.5–7.1)	0.498 (− 0.2 to 0.5)	6.6 (6.0–7.3)	**0.006** (− 0.4 to − 0.7)
Missing values, n (%)	65 (16.5)	31 (20.2)	34 (14.0)		2 (2.9)	
**Thrombocytes**, median in per 10^9^/L, (IQR)	141.0 (95.0–372.0)	150.5 (91.5–455.2)	140.0 (95.5–317.5)	0.556 (− 18.7 to 34.7)	464.0 (258.0–642.0)	** < 0.001** (− 415.8 to − 230.2)
Missing values, n (%)	69 (17.5)	32 (20.9)	37 (15.3)		5 (7.2)	
**Leukocytes**, median in per 10^9^/L, (IQR)	12.5 (5.3–19.4)	12.8 (5.1–19.6)	12.3 (5.4–19.0)	0.762 (− 2.7 to 3.7)	14.8 (6.9–18.0)	0.202 (− 5.3 to 1.1)
Missing values, n (%)	83 (21.0)	32 (20.9)	51 (21.1)		4 (5.8)	
**Neutrophils**, median in per 10^9^/L, (IQR)	6.7 (3.1–12.1)	7.5 (2.5–12.8)	6.4 (3.4–11.4)	0.419 (− 1.5 to 3.7)	0.91 (0.001–9.8)	**0.002** (2.1–9.0)
Missing values, n (%)	265 (67.1)	101 (66.0)	164 (67.8)		32 (46.4)	
**Lymphocytes**, median in per 10^9^/L, (IQR)	0.80 (0.42–2.1)	0.78 (0.48–1.5)	0.90 (0.40–2.5)	0.512 (− 0.5 to 3.7)	0.50 (0.18–2.5)	0.564 (− 0.7 to 1.3)
Missing values, n (%)	264 (66.8)	101 (66.0)	163 (67.4)		35 (50.7)	
**C-reactive protein**, median in mg/L, (IQR)	187.8 (118.1–253.4)	174 (119.7–258.5)	196.5 (116.5–251.8)	0.075 (− 1.5 to 54.1)	122 (63.7–176.0)	** < 0.001** (35.3–97.7)
Missing values, n (%)	27 (6.8)	11 (7.2)	16 (6.6)		2 (2.9)	
**Ferritin**, median in ng/mL, (IQR)	508 (296.8–883.5)	537 (297.0–839.3)	485 (295.0–883.5)	0.322 (− 170.6 to 57.3)	211.5 (155.9–318.8)	** < 0.001** (236.7–379.8)
Missing values, n (%)	115 (29.1)	45 (29.4)	70 (28.9)		29 (42.0)	
**NT-proBNP**, median in pmol/L, (IQR)	556 (130.4–1436.5)	435.1 (101.3–1431.5)	585.7 (151.9–1434.5)	**0.015** (− 78.4 to 424.7)	151.4 (52.9–331.8)	** < 0.001** (287.5–575.4)
Missing values, n (%)	99 (25.1)	38 (24.8)	61 (25.2)		27 (39.1)	
**Troponin T**, median in µg/L, (IQR)	44.8 (16.6–175.1)	77.2 (20.6–268.5)	38.7 (16–0–144.5)	0.011 (− 73.8 to 8.9)	8.0 (4.9–29.0)	** < 0.001** (25.1–49.9)
Missing values, n (%)	104 (26.3)	42 (27.5)	62 (25.6)		30 (43.5)	
**D dimer**, median in mg/FEU, (IQR)	4.3 (2.4–8.5)	4.0 (2.2–7.6)	4.4 (2.6–8.6)	0.079 (− 0.6 to 1.1)	2.0 (1.3–4.2)	**0.014** (1.3–3.8)
Missing values, n (%)	95 (24.1)	34 (22.2)	61 (25.2)		29 (42.0)	
**GOT**, median in U/L, (IQR)	45.0 (30.0–77.2)	48.0 (31.5–88.9)	43.0 (29.0–72.0)	0.417 (− 7.1 to 17.1)	39.0 (25.0–79.5)	0.388 (− 7.7 to 19.7)
Missing values, n (%)	128 (32.4)	57 (37.3)	71 (29.3)		11 (15.9)	
**GPT**, median in U/L, (IQR)	42.0 (22.0–67.0)	48.0 (21.0–87.0)	38.7 (23.2–59.0)	0.264 (− 6.1 to 22.1)	42.0 (20.5–138.2)	1.000 (− 20.5 to 20.5)
Missing values, n (%)	108 (27.3)	46 (30.1)	62 (25.6)		10 (14.5)	
**Bilirubin, total**, median in µmol/L, (IQR)	8.6 (5.1–15.4)	9.1 (5.3–15.4)	8.6 (5.1–15.4)	0.571 (− 2.6 to 4.7)	6.8 (3.4–20.3)	0.531 (− 3.7 to 7.1)
Missing values, n (%)	221 (55.9)	94 (61.4)	127 (52.5)		39 (56.5)	
**Albumin**, median in g/dL, (IQR)	2.7 (2.2–3.3)	2.7 (2.2–3.2)	2.7 (2.3–3.3)	1.000 (− 0.2 to 0.2)	2.9 (2.5–3.7)	0.145 (− 0.5 to 0.1)
Missing values, n (%)	138 (34.9)	64 (41.8)	74 (30.6)		17 (24.6)	
**Creatinine**, median in µmol /L, (IQR)	46.9 (34.5–70.7)	49.5 (33.6–79.6)	46.0 (35.4–64.9)	0.434 (− 5.3 to 12.3)	26.5 (23.4–35.4)	** < 0.001** (16.4–25.3)
Missing values, n (%)	121 (30.6)	53 (34.6)	68 (28.1)		11 (15.9)	
**SARS-CoV-2 PCR positive**, n (%)	54 (13.7)	28 (18.3)	26 (10.7)	**0.030** (− 14.5 to 0.6)	0	** < 0.001** (5.5–21.8)
**SARS-CoV-2 Antigen positive**, n (%)	13 (3.3)	6 (3.9)	7 (2.9)	0.580 (− 4.7 to 2.6)	0	0.130 (− 0.9 to 7.5)
**SARS-CoV-2 S1 antibody positive**, n (%)	338 (85.6)	125 (81.7)	213 (88.0)	0.080 (− 0.8 to 13.4)	0	** < 0.001** (77.2–93.9)
**SARS-CoV-2 contacts positive**, n (%)	3 (0.8)	0	3 (1.2)	n.d	0	n.d

PIMS, pediatric multisystem inflammatory syndrome; KD, Kawasaki disease. ^a^comparison between PIMS-non-KD and PIMS-KD. ^b^comparison between PIMS-all and KD; 95% CI, 95%-confidence interval; n; number of cases; n.sh., not shown due to expected differences in case definitions; pARDS, pediatric acute respiratory distress syndrome; IQR, interquartile range; PCR, polymerase chain reaction; n.d.; not determined. *P* values in bold are considered statistically significant.

In 38% and 39% of cases, respectively, PIMS-all and KD were correctly diagnosed upon admission (Supplementary Table 2). Among PIMS-all cases, the most important differential diagnoses upon admission were gastroenteritis, fever of unknown origin, sepsis, acute appendicitis and KD. Among KD patients, gastroenteritis and acute appendicitis were less commonly considered as potential differential diagnoses.

### Laboratory characteristics

No specific laboratory marker exists for either PIMS or KD. As compared to KD, however, PIMS-all patients had higher values for neutrophils, CRP, ferritin, creatinine, NT-proBNP, troponin T, and d-dimers as well as lower values for hemoglobin, and thrombocytes (Table 2). By contrast, PIMS-KD showed higher titers for NT-proBNP and d-dimers than did PIMS-non-KD patients.

### Treatment

Median length of hospitalization was similar between PIMS-all (10d [IQR 8–12]) and KD cases (8d [IQR 6–12]), without there being any difference between PIMS-non-KD and PIMS-KD patients (Table 3). Over 90% of PIMS-all patients received a PIMS-directed therapy during their hospitalizations. Most commonly, this included immunomodulatory medication (89%), followed by systemic antibiotics (71%), hemostaseological medication (51%), pulmonary support (32%), and inotropes/vasodilators (28%).

**Table 3:** Therapy and outcome in patients with PIMS-all, PIMS-non-KD, PIMS-KD and KD.

	PIMS-all (n = 395)	PIMS-non-KD (n = 153)	PIMS-KD (n = 242)	*p*-value^a^ (95% CI)	KD (n = 69)	*p*-value^b^ (95% CI)
**Any therapy**, n (%)	364 (92.2)	139 (90.8)	225 (93.0)	0.450 (− 3.3 to 7.6)	57 (82.6)	**0.010** (2.1–16.9)
Duration of hospitalization, median in days (IQR)	10 (8–12)	9 (7–13)	10 (8–12)	0.545 (0.2–2.4)	8 (6–12)	0.113 (0.7–3.6)
Missing values, n (%)	72 (18.2)	33 (21.6)	39 (16.1)		9 (13.0)	
**ICU**, n (%)	190 (48.1)	76 (49.7)	114 (47.1)	0.620 (− 12.7 to 7.6)	13 (18.8)	** < 0.001** (16.8–41.7)
Duration of ICU, median in days (IQR)	1 (0–5)	2 (0–6)	0 (0–5)	**0.008** (− 5.6 to 0.0)	0 (0–0)	0.731 (− 0.3 to 2.8)
Missing values, n (%)	37 (9.4)	18 (11.8)	19 (7.9)		6 (8.7)	
**Pulmonary support**, n (%)	126 (31.9)	56 (36.6)	70 (28.9)	0.110 (− 17.1 to 1.8)	7 (10.1)	**0.000** (10.3–33.2)
O_2_-supplementation, n (%)	110 (27.8)	47 (30.7)	63 (26.0)	0.310 (− 13.8 to 4.4)	7 (10.1)	** < 0.001** (6.7–28.7)
High-flow, n (%)	38 (9.6)	19 (12.4)	19 (7.9)	0.130 (− 10.6 to 1.4)	1 (1.4)	**0.020** (1.1–15.3)
Continuous pulmonary airway pressure, n (%)	13 (3.3)	11 (7.2)	2 (0.8)	** < 0.001** (− 9.9 to 2.8)	0	0.130 (− 0.9 to 7.5)
Invasive ventilation, n (%)	48 (12.2)	27 (17.6)	21 (8.7)	**0.010** (− 15.6 to 2.4)	1 (1.4)	**0.010** (2.9–18.5)
**Inotropes/Vasodilators**, n (%)	111 (28.1)	42 (27.5)	69 (28.5)	0.820 (− 8.1 to 10.2)	2 (2.9)	** < 0.001** (14.4–36.0)
**Hemo-/Peritoneal dialysis**, n (%)	1 (0.3)	1 (0.7)	0	0.210 (− 1.7 to 0.4)	0	0.680 (− 0.9 to 1.4)
**Antivirals**, n (%)	3 (0.8)	2 (1.3)	1 (0.4)	0.320 (− 2.7 to 0.9)	0	0.470 (− 1.3 to 2.8)
**Antibiotics, systemic**, n (%)	280 (70.9)	103 (67.3)	177 (73.1)	0.220 (− 3.4 to 15.1)	46 (66.7)	0.480 (− 7.5 to 16.0)
**Immunomodulators**, n (%)	352 (89.1)	129 (84.3)	223 (92.1)	**0.010** (1.5–14.1)	61 (88.4)	0.860 (− 7.3 to 8.7)
IVIG, n (%)	322 (81.5)	114 (74.5)	208 (86.0)	** < 0.001** (3.6–19.3)	55 (79.7)	0.720 (− 8.2 to 11.8)
IVIG-only, n (%)	52 (13.2)	20 (13.1)	32 (13.2)	0.970 (− 6.7 to 7.0)	17 (24.6)	**0.010** (− 20.6 to 2.4)
Corticosteroids, n (%)	262 (66.3)	98 (64.1)	164 (67.8)	0.450 (− 5.9 to 13.3)	36 (52.2)	**0.020** (1.9–26.4)
Corticosteroids-only, n (%)	118 (29.9)	52 (34.0)	66 (27.3)	0.160 (− 16.0 to 2.6)	16 (23.2)	0.260 (− 4.9 to 18.3)
IVIG + corticosteroids, n (%)	234 (59.2)	83 (54.2)	151(62.4)	0.110 (− 1.8 to 18.1)	32 (46.4)	0.050 (0.2–25.5)
Anti-IL-1 inhibitors, n (%)	7 (1.8)	3 (2.0)	4 (1.7)	0.820 (− 3.0 to 2.4)	0	0.270 (− 1.4 to 4.9)
Anti-IL-6 inhibitors, n (%)	3 (0.8)	1 (0.7)	2 (0.8)	0.850 (− 1.6 to 1.9)	0	0.470 (− 1.3 to 2.8)
Anti-TNFalpha inhibitors, n (%)	2 (0.5)	0	2 (0.8)	0.260 (− 0.6 to 2.3)	1 (1.4)	0.370 (− 3.0 to 1.1)
**Hemostaseological**, n (%)	200 (50.6)	73 (47.7)	127 (52.5)	0.360 (− 5.4 to 14.9)	32 (46.4)	0.520 (− 8.6 to 17.1)
Heparin, n (%)	85 (21.5)	40 (26.1)	45 (18.6)	0.080 (− 15.9 to 0.8)	2 (2.9)	** < 0.001** (8.7–28.5)
Acetylsalicylic-acid, n (%)	169 (42.8)	53 (34.6)	116 (47.9)	**0.010** (3.3–23.3)	30 (43.5)	0.910 (− 13.4 to 12.0)
**Transfusion**, n (%)	25 (6.3)	15 (9.8)	10 (4.1)	**0.020** (− 10.6 to 0.7)	2 (2.9)	0.260 (− 2.6 to 9.4)
**Other**, n (%)	61 (15.4)	31 (20.3)	30 (12.4)	**0.040** (− 15.2 to 0.5)	7 (10.1)	0.250 (− 3.8 to 14.4)
**Outcome**						
Restitutio ad integrum, n (%)	210 (53.2)	88 (57.5)	122 (50.4)	0.170 (− 17.2 to 3.0)	37 (53.6)	0.940 (− 13.3 to 12.4)
Symptoms (potentially reversible) at discharge, n (%)	167 (42.3)	60 (39.2)	107 (44.2)	0.330 (− 5.0 to 15.0)	27 (39.1)	0.630 (− 9.5 to 15.8)
Respiratory tract symptoms, n (%)	15 (3.8)	10 (6.5)	5 (2.1)	**0.020** (− 8.3 to 0.6)	1 (1.4)	0.330 (− 2.3 to 7.0)
Cardiovascular symptoms, n (%)	86 (21.8)	27 (17.6)	59 (24.4)	0.110 (− 1.6 to 15.1)	18 (26.1)	0.430 (− 15.0 to 6.4)
Gastrointestinal symptoms, n (%)	10 (2.5)	7 (4.6)	3 (1.2)	**0.040** (− 6.5 to 0.2)	0	0.180 (− 1.2 to 6.3)
Hepatic symptoms, n (%)	6 (1.5)	4 (2.6)	2 (0.8)	0.160 (− 4.3 to 0.7)	0	0.300 (− 1.4 to 4.4)
Renal symptoms, n (%)	2 (0.5)	0	2 (0.8)	0.260 (− 0.6 to 2.3)	0	0.550 (− 1.2 to 2.2)
Neurological symptoms, n (%)	12 (3.0)	3 (2.0)	9 (3.7)	0.320 (− 1.7 to 5.2)	0	0.140 (− 1.0 to 7.1)
Hematological symptoms, n (%)	8 (2.0)	3 (2.0)	5 (2.1)	0.940 (− 2.8 to 3.0)	0	0.230 (− 1.3 to 5.4)
Skin changes, n (%)	19 (4.8)	5 (3.3)	14 (5.8)	0.260 (− 1.8 to 6.9)	3 (4.8)	0.870 (− 5.0 to 5.9)
Mucosal changes, n (%)	11 (2.8)	2 (1.3)	9 (3.7)	0.160 (− 0.9 to 5.8)	0	0.160 (− 1.1 to 6.7)
Sequelae (potentially irreversible) at discharge, n (%)	28 (7.1)	5 (3.3)	23 (9.5)	**0.020** (1.0–11.4)	8 (11.6)	0.200 (− 11.4 to 2.4)
Cardiovascular sequelae, n (%)	27 (6.8)	5 (3.3)	22 (9.1)	**0.030** (0.7–10.9)	8 (11.6)	0.170 (− 11.5 to 2.0)
Cardiac failure, n (%)	3 (0.8)	0	3 (1.2)	0.170 (− 0.5 to 3.0)	0	0.470 (− 1.3 to 2.8)
Coronary artery aneurysm, n (%)	15 (3.8)	2 (1.3)	13 (5.4)	**0.040** (0.2–7.9)	5 (7.2)	0.190 (− 8.7 to 1.8)
Other sequelae, n (%)	1 (0.3)	0	1 (0.4)	0.430 (− 0.6 to 1.4)	0	0.680 (− 0.9 to 1.4)
Death, n (%)	0	0	0	n.a	0	n.a

PIMS, Pediatric Multisystem Inflammatory Syndrome; KD, Kawasaki disease. ^a^comparison between PIMS-non-KD and PIMS-KD. ^b^comparison between PIMS-all and KD; 95% CI, 95%-confidence interval; n; number of cases; IQR, interquartile range; ICU, intensive care unit; IVIG, intravenous immunoglobulins; n.a., not applicable. *P* values in bold are considered statistically significant.

Overall, PIMS-all were more severely ill than KD patients, as indicated by a higher rate of ICU admission (48% vs. 19%), longer ICU stays (1 day [IQR 0–5] vs. < 0.5 day [IQR 0– < 0.5]), pulmonary support (32% vs. 10%), including invasive ventilation (12% and 1%, respectively), and inotropes/vasodilators (28% vs. 3%) (Table 3). Similarly, PIMS-non-KD were more severely affected than were PIMS-KD patients, (longer duration of ICU stay, and higher rates of continuous positive airway pressure (CPAP), ventilation and transfusion). PIMS-KD patients more often received IVIG and acetylsalicylic acid (ASA), and less commonly were prescribed heparin and corticosteroids.

### Outcome

Among both PIMS-all and KD patients, the overall outcomes were comparably positive (Table 3). Significantly, no patient died. At discharge, 40% of patients, (both PIMS-all and KD), continued to have symptoms considered reversible by the reporting physician. Sequelae reported—almost exclusively cardiovascular—were present in 7% of PIMS-all and 12% of KD patients. The most common cardiovascular sequelae were cardiac insufficiency (n = 3 in PIMS-all vs. n = 0 in KD) and coronary artery dilatation or coronary artery aneurysms (CAA) (n = 15 in PIMS-all vs. n = 5 in KD). PIMS-KD more frequently were affected by coronary artery dilatation or CAA than were PIMS-non-KD patients (n = 13 vs. n = 2).

## Discussion

Our national registry is one of the largest collections of PIMS cases worldwide. It also is the only dataset collecting data in parallel from both SARS-CoV-2-associated PIMS and non-SARS-CoV-2 KD cases. By contrast, other studies comparing PIMS and KD patients have used historical controls^10,11,16^. In our analysis of the PIMS and KD cases in this cohort, clinical and epidemiological similarities, as well as differences, became clear. There were more PIMS-all than KD cases reported to the registry (5.7:1 ratio). Reporting of PIMS-all cases climaxed five to seven weeks following a peak in hospitalized COVID-19 cases (Fig. 1). This pattern is well-known in PIMS and typically occurs two to eight weeks following a COVID-19 surge^12,17–19^. Beginning in May 2021, the reporting of KD cases to our registry decreased; reporting of KD cases has continued to remain low since then, although the reason for this lower-level reporting is not fully understood. Given that reporting to this national PIMS and KD registry is voluntarily, a reporting bias cannot be excluded. The overall numbers of KD cases reported is lower than that reported in a population-based epidemiological study from Germany in 2011–2012^20^. During 2011–2012, 315 KD cases were submitted within a 24-month period^20^, whereas during an 18-month period in 2020–2021, only 69 cases were documented in the registry.

The male-to-female ratio among PIMS-all and KD cases was comparable, with a higher number of male cases (1.8:1 vs. 1.7:1; Table 1). This also has been reported in other cohorts from other countries^7–11,16,18^. In our cohort, only one in four PIMS cases and one in five KD cases had a concomitant disease. No singular comorbidity stood out. By contrast, in the United States, a larger proportion of PIMS cases (38%) had underlying conditions, most commonly obesity and chronic lung disease^19^. Coincidentally, among KD cases in a US cohort, the rate of concomitant diseases was lower than in PIMS cases^16^.

In our cohort, PIMS and KD patients displayed several key epidemiological and clinical differences. PIMS-KD cases were more commonly incomplete (67%) than KD cases (46%). Similar findings have not been previously reported. In KD, incomplete cases more commonly affected younger patients—a factor often leading to delays in diagnosis and a poorer cardiac prognosis^21^. PIMS-all patients generally were older than KD patients (median of 7 vs. 3 years); among PIMS-all, PIMS-KD patients were younger than PIMS-non-KD patients (median of 7 vs. 9 years). This PIMS vs. KD age difference has been well-documented in the literature^7–11,16,22^.

The clinical presentation of PIMS and KD differed as well. Whereas PIMS-all patients more commonly experienced gastrointestinal (including ascites), cardiovascular, respiratory, renal and neurological symptoms, KD patients more commonly presented with signs and symptoms of cutaneous, mucosal, cervical lymphadenopathy, and ear-nose-throat systems, as well as arthritis and arthralgia. A logistic regression analysis demonstrated the differences between PIMS and KD regarding gastrointestinal symptoms, hematological symptoms and arthritis remained significant (Supplementary Table 3). We observed the same preferential organ involvement in PIMS-KD as compared to PIMS-non-KD. The differences in cutaneous involvement, mucosal involvement and cervical lymphadenopathy are due to a difference in case definitions. Among PIMS-non-KD cases, dyspnea/tachypnea, pneumonia, pARDS on X-ray, thickening of enteric walls on ultrasound and nephritis were symptoms/signs that allowed us to distinguish it from PIMS-KD. By contrast, PIMS-KD cases more commonly presented with vomiting and hepatosplenomegaly.

Although no single laboratory biomarker specifically allows us to distinguish between PIMS-all and KD, inflammatory markers (such as the acute phase proteins CRP and ferritin), the pro-coagulant d-dimer, and cardiac function markers (such as proBNP and troponin T/I) are elevated in both PIMS and KD^12,23^. In our cohort, the serum concentration of these markers was higher in PIMS-all than it was in KD. A linear regression analysis showed that the differences between PIMS and KD regarding proBNP levels remained significant (Supplementary Table 4). Surprisingly however, d-dimer and proBNP values were higher in PIMS-KD than in PIMS-non-KD, indicating a higher degree of inflammation.

Overall, PIMS patients displayed more severe symptoms than KD patients. PIMS-all cases were admitted to the ICU more frequently and needed greater respiratory and circulatory support than did KD patients. This finding was confirmed by logistic regression analysis (Supplementary Table 5). PIMS-all patients also more often were treated with steroids and heparin and less often with IVIG alone. A linear regression analysis proved that the differences between PIMS and KD regarding corticosteroid and heparin treatments stayed significant (Supplementary Table 5). PIMS-KD were admitted to the ICU less often and required less CPAP and invasive ventilation than did PIMS-all cases; they were given IVIG and ASA more frequently. What role the addition of corticosteroids to IVIG may play in PIMS or KD is open to debate. To date, no randomized clinical trials have been performed for pediatric PIMS^23,24^. In patients with cardiovascular failure and shock, corticosteroid treatment is recommended^22,23^. By contrast, in ACR’s 2022 treatment guidelines, corticosteroid treatment in addition to IVIG is recommended for all hospitalized PIMS patients^25^. The use of antibiotics in our cohort was high (71% in PIMS-all vs. 67% in KD). In a review of case series, rates between 67 and 100% were reported^26^. This high prescription rate is not surprising, because at presentation, bacterial sepsis is an important differential diagnosis in both PIMS and KD patients and empirical antibiotic treatment is recommended in PIMS until the point when bacterial cultures come back negative^22^.

The overall outcome of PIMS and KD is favorable. Most patients in our cohort had completely recovered upon discharge. To date, we have had no reports of patients who died of PIMS-all or KD. Mortality in resource-rich countries is reported to be < 1–2%^19,24,27^. Sequelae considered irreversible by the reporting physician at time of patient discharge mainly were related to cardiovascular symptoms (in 7% in PIMS-all vs. 12% in KD). These numbers were similar between PIMS-all and KD both in bivariate and multiple regression analysis (Supplementary Table 6). It was only among PIMS-KD patients that the rate of developing CAA at discharge was higher than among PIMS-non-KD cases. CAAs in PIMS are usually small (z-score < 5^24^) and the prognosis is considered more favorable than in KD^13,23,24^. Most of the CAAs have resolved within six months following the acute PIMS phase^28,29^. However, fatigue and exercise intolerance were reported among up to 50% of PIMS cases upon six-month-follow-up^18,29^. This suggests that a structured follow-up is particularly warranted for PIMS and KD patients. In Germany, the register for acute PIMS cases will be supplemented by a follow-up register designed to collect data that will allow us to better understand the long-term prognosis of PIMS. In addition, we plan to analyze data collected during the fourth COVID-19 wave in late 2021 (one dominated by the Delta variant) and the fifth wave in early 2022 (dominated by the Omicron variant), so as to determine whether these variants have altered the clinical picture of PIMS in Germany.

The particular strength of our data lies in its concomitant compilation of PIMS and KD cases in a nationwide registry. This gives us the ability to compare these two entities for the same time period without needing to employ historic controls. This data previously has not been available. The main limitation of our study lies in its voluntary reporting registry design—an approach that may lead to selection bias. Because, however, a COVID-19 registry collecting data from hospitalized acute COVID-19 cases accurately mirrored data trends shown in the statutory notification system^30^, we nevertheless are confident that our data is representative of the PIMS epidemiological situation in Germany. PIMS is not a notifiable disease in Germany; therefore, no notification data for it exist as such. Due to the nature of PIMS and its severe symptoms, however, underreporting seems even less likely than it would be for acute hospitalized COVID-19 cases. Another factor limiting the interpretation of therapies on outcome is the fact that we did not gather information on the timing of therapy for either PIMS or KD patients.

Despite differences in age distribution and disease severity, widely-shared clinical characteristics and a similar prognosis suggest that PIMS and KD represent a syndrome continuum based upon hyperinflammation triggered by an infectious agent. Follow-on studies of SARS-CoV-2-induced hyperinflammation will help generating hypotheses regarding the etiology of KD.

## Conclusion

In Germany, PIMS and KD cases appear to display more clinical and prognostic similarities than they do differences. This suggests that they represent points on a syndrome continuum rather than separate diseases per se.

## Methods

On March 18, 2020, a prospective registry for children and adolescents hospitalized with PIMS in German pediatric hospitals was established. The study was approved by the Ethics Committee of the Technische Universität Dresden (BO-EK-110032020) and was assigned clinical trial number DRKS00021506. All methods were performed in accordance with the relevant guidelines and regulations (https://www.akek.de/sonstige-studien/). The need for informed consent was waived by the institutional review board of the Ethics Committee of the Technische Universität Dresden (BO-EK-110032020).

### Patients and setting

All German pediatric hospitals were invited to participate by prospectively reporting PIMS and KD cases. For each patient, an electronic case report form was completed in a secure database, with the link accessible through the DGPI website (https://dgpi.de/pims-survey-anleitung/). Included were patients under 20 years old who had been hospitalized with PIMS/KD during the period March 18, 2020–August 31, 2021. Overall, 517 patients were reported to the registry between March 2020 and August 2021. Data collected included demographic characteristics, comorbidities, initial symptoms and clinical signs, laboratory and imaging tests, treatments, disease course during hospitalization, and outcome at hospital discharge. All PIMS and KD cases reported were reviewed by two of the study’s authors (J.A., M.H.) in order to verify whether the cases fulfilled the WHO criteria for PIMS^5^ and/or the American Heart Association criteria for KD^14^. Organ involvement in PIMS cases was defined according to WHO criteria^5^. Whenever potential differences of opinion emerged, additional information was collected from the reporting physician, discussed within the core team (J.A., R.B., M.H.) and subsequently categorized. PIMS cases were required either to have a positive SARS-CoV-2 serology or -PCR, or else be known to have had close contact with a SARS-CoV-2-infected person. KD cases were required to be either SARS-CoV-2 serology-negative or PCR-negative, without a known close contact with SARS-CoV-2 infection. PIMS patients (labeled PIMS-all) were categorized into two groups: (1) PIMS *without* features of KD (labeled PIMS-non-KD) and (2) PIMS *with* features of KD (labeled PIMS-KD), if the patient had at least two classical KD features. This differentiation was chosen based on an early report on an Italian PIMS cohort^15^ where the authors observed a high rate of intravenous immunoglobulin G (IVIG) resistance in PIMS patients without KD features and therefore suggested that glucocorticoids (in addition to IVIG) may play a role in this PIMS subgroup^15^. PIMS-KD and KD patients were additionally divided into the groups “complete KD” (with 4–5 KD features) or “incomplete KD” (with just 2–3 KD features). Information on the following comorbidity groupings was gathered: respiratory, cardiovascular, gastrointestinal, hepatic, renal, neurological/neuromuscular, psychiatric, hematological, oncological, s/p transplant (solid organ or bone marrow), autoimmune and immunodeficiency (including immunosuppressive treatment). The main outcome categories tracked upon patient discharge were: restitutio ad integrum (i.e., asymptomatic at discharge), persistent symptoms (potentially reversible symptoms at discharge), sequelae at discharge (potentially irreversible symptoms at discharge), and case fatality.

### Statistical analysis

For statistical analysis, Microsoft Excel v.2010 and the software programs IBM SPSS v.25.0 and R v3.6. were employed.

Based upon the presence or absence of KD features, the following comparisons were conducted: (A) patients with PIMS and Kawasaki (PIMS-KD) vs. patients with PIMS without Kawasaki (PIMS-non-KD); and (B) patients with PIMS (both with and without Kawasaki [PIMS-all]) vs. patients with Kawasaki without SARS-CoV-2 infection (KD).

Sociodemographic characteristics were described by absolute and relative frequencies for categorical variables, while continuous variables were described by median and first/third quartile. Group differences among these variables were tested using Fisher's exact test for categorical variables and Wilcoxon rank-sum test for continuous variables. In addition, group differences were assessed with respect to preliminary diagnoses on admission, comorbidities, symptoms, diagnostic procedures, laboratory findings, therapies and outcomes. Differences regarding the probability of occurrence in the event of binary variables, as well as regarding median values in the event of continuous variables, were estimated. Confidence intervals and *p*-values were derived by the Wald method for differences in probabilities, as well as by the bootstrap method with 1000 replications for differences in median values. To adjust group differences between KD and PIMS-all patients on the basis age and sex, we used linear regression analysis for continuous outcomes and logistic regression analysis for binary outcomes. The significance and confidence levels were set to 0.05 and 0.95, respectively.

## Supplementary Information

Supplementary Information.

## Data Availability

The data presented in this study are available on reasonable request from the corresponding author. The data are not publicly available due to ethical and data privacy protection obligations.

## Abbreviations

ACR: American College of Rheumatology
AHA: American Heart Association
ASA: Acetylsalicylic Acid
CAA: Coronary Artery Aneurysm(s)
CDC: Centers of Disease Control
CI: Confidence Interval
COVID-19: Coronavirus Disease 2019
CPAP: Continuous Positive Airway Pressure
CRP: C-Reactive Protein
DGPI: Deutsche Gesellschaft für Pädiatrische Infektiologie (German Society for Pediatric Infectious Diseases)
FUO: Fever of Unknown Origin
ICU: Intensive Care Unit
IQR: Interquartile Range
IVIG: Immunoglobulin G
KD: Kawasaki Disease
MIS-C: Multisystem Inflammatory Syndrome in Children
NT-proBNP: N-terminal pro Brain Natriuretic Protein
pARDS: Pediatric Acute Respiratory Distress Syndrome
PCR: Polymerase Chain Reaction
PID: Primary Immunodeficiency
PIMS: Pediatric Inflammatory Multisystem Syndrome
PIMS-KD: Pediatric Inflammatory Multisystem Syndrome *with* Kawasaki Disease features
PIMS-non-KD: Pediatric Inflammatory Multisystem Syndrome *without* Kawasaki Disease features
PIMS-TS: Pediatric Inflammatory Multisystem Syndrome temporarily-associated with SARS-CoV-2
SARS-CoV-2: Severe Acute Respiratory Syndrome Coronavirus type 2
s/p: Status post
TSS: Toxic Shock Syndrome
vs.: Versus
WHO: World Health Organization
