# Supplementary material for: A comparison of pediatric inflammatory multisystem syndrome temporarily-associated with SARS-CoV-2 and Kawasaki disease
Source: Sci Rep. 2023 Jan 20;13:1173. doi: 10.1038/s41598-022-26832-5 (PMC9857913; doi:10.1038/s41598-022-26832-5)
Supplement: Supplementary file 1 — Supplementary Information. [file 41598_2022_26832_MOESM1_ESM.docx]

| **Diagnosis of cases excluded from the analysis** | **Number of cases** |
| --- | --- |
| SARS-CoV-2 associated inflammatory diseases (pericarditis, systemic juvenile arthritis-like) | 2 |
| COVID-19 disease | 13 |
| Non-SARS-CoV-2 associated inflammatory disease (pericarditis, systemic juvenile arthritis, HLH) | 9 |
| Other infections (sepsis, bacteremia, Toxic-shock syndrome, adenovirus infection, other systemic viral infections) | 28 |
| Unknown diseases (incomplete case information) | 1 |

**Supplemental Table 1:** Diagnosis of cases that fulfilled neither PIMS nor KD criteria and that were excluded from analysis.

PIMS; Pediatric Multisystem Inflammatory Syndrome; KD, Kawasaki disease; HLH; hemophagocytic lymphohistiocytosis

|  | PIMS-all  (n=395) | PIMS-non-KD (n=153) | PIMS-KD (n=242) | p-value^a^ | KD  (n=69) | p-value^b^ |
| --- | --- | --- | --- | --- | --- | --- |
| **Suspected diagnosis on admission***  Upper respiratory tract infection, n (%)  Bronchitis, n (%)  Pneumonia, n (%)  Acute respiratory distress syndrome, n (%)  Gastroenteritis, n (%)  Appendicitis, n (%)  Peritonitis, n (%)  Pancreatitis, n (%)  Meningitis, n (%)  Encephalitis, n (%)  Sepsis, n (%)  Systemic inflammatory response syndrome, n (%)  Disseminated intravascular coagulation, n (%)  Myocarditis, n (%)  Pediatric inflammatory multisystem syndrome, n (%)  Kawasaki disease, n (%)  Fever of unknown origin, n (%)  COVID-19 infection, n (%)  Other diseases, n (%) | 36 (9.1)  2 (0.5)  15 (3.8)  6 (1.5)  96 (24.3) 43 (10.9)  12 (3.0)  1 (0.3)  21 (5.3)  4 (1.0)  61 (15.4)  1 (0.3)  1 (0.3)  7 (1.8)  148 (37.5)  43 (10.9)  72 (18.2)  12 (3.0)  53 (13.4) | 14 (9.2)  1 (0.7)  6 (3.9)  3 (2.0)  36 (23.5) 22 (14.4)  7 (4.6)  1 (0.7)  10 (6.5)  3 (2.0)  29 (19.0)  1 (0.7)  1 (0.7)  3 (2.0)  56 (36.6)  2 (1.3)  31 (20.3)  5 (3.3)  21 (13.7) | 22 (9.1)  1 (0.4)  9 (3.7)  3 (1.2)  60 (24.8) 21 (8.7)  5 (2.1)  0  11 (4.5)  1 (0.4)  32 (13.2)  0  0  4 (1.7)  92 (38.0)  41 (16.9)  41 (16.9)  7 (2.9)  32 (13.2) | 0.980  0.740  0.920  0.570  0.780  0.080  0.160  0.210  0.390  0.140  0.130  0.210  0.210  0.820  0.780  0.000  0.410  0.830  0.890 | 9 (13.0)  1 (1.4)  3 (4.3)  0  6 (8.7)  1 (1.4)  0  0  3 (4.3)  0  9 (13.0)  0  0  0  10 (14.5)  27 (39.1)  23 (33.3)  2 (2.9)  14 (20.3) | 0.310  0.370  0.830  0.300  0.000  0.010  0.140  0.680  0.740  0.400  0.610  0.680  0.680  0.270  0.000  0.000  0.000  0.950  0.130 |

**Supplemental Table 2**: Suspected diagnosis on admission of patients with PIMS-all, PIMS-non-KD, PIMS-KD and KD.

*Multiple suspected diagnosis on admission were reported; PIMS, Pediatric Multisystem Inflammatory Syndrome; KD, Kawasaki disease; ^a^comparison between PIMS-non-KD and PIMS-KD; ^b^comparison between PIMS-all and KD

|  | **OR** | **95%-CI** | **p-value** |
| --- | --- | --- | --- |
| **Testing for cardiac symptoms** | | | |
| Comparison PIMS-all vs. KD | 1.612 | 0.912-2.852 | 0.101 |
| **Testing for respiratory symptoms** | | | |
| Comparison PIMS-all vs. KD | 1.640 | 0.927-2.903 | 0.089 |
| **Testing for gastrointestinal symptoms** | | | |
| Comparison PIMS-all vs. KD | 2.328 | 1.251-4.003 | **0.007** |
| **Testing for renal symptoms** | | | |
| Comparison PIMS-all vs. KD | 1.894 | 0.808-4.441 | 0.142 |
| **Testing for neurological symptoms** | | | |
| Comparison PIMS-all vs. KD | 1.917 | 0.855-4.298 | 0.114 |
| **Testing for arthritis** | | | |
| Comparison PIMS-all vs. KD | 0.203 | 0.051-0.806 | **0.023** |
| **Testing for hematological symptoms** | | | |
| Comparison PIMS-all vs. KD | 2.395 | 1.262-4.546 | **0.008** |

**Supplemental Table 3**: Comparison of specific organ manifestations between PIMS-all and KD patients, using logistic regression modelling.

OR, odds ratios; 95%-CI, 95%-confidence interval; Pediatric Multisystem Inflammatory Syndrome; KD, Kawasaki disease

|  | **Beta** | **95%-CI** | **p-value** |
| --- | --- | --- | --- |
| **Testing for CRP** | | | |
| Comparison PIMS-all vs. KD | -1205.7 | -3790.0 – 1378.7 | 0.361 |
| **Testing for lymphocytes** | | | |
| Comparison PIMS-all vs. KD | 371.7 | -21253.3 – 21996.7 | 0.973 |
| **Testing for NT-proBNP** | | | |
| Comparison PIMS-all vs. KD | 809.2 | 271.4 – 1347.0 | **0.003** |
| **Testing for troponin T** | | | |
| Comparison PIMS-all vs. KD | 7856.2 | -14189.1 – 29901.5 | 0.485 |
| **Testing for albumin** | | | |
| Comparison PIMS-all vs. KD | -549.1 | -2505.0 – 1406.7 | 0.583 |

**Supplemental Table 4**: Comparison of important laboratory values between PIMS-all and KD patients, using linear regression modelling.

Beta, differences of median; 95%-CI, 95%-confidence interval; Pediatric Multisystem Inflammatory Syndrome; KD, Kawasaki disease

|  | **OR** | **95%-CI** | **p-value** |
| --- | --- | --- | --- |
| **Testing for ICU treatment** | | | |
| Comparison PIMS-all vs. KD | 2.851 | 1.471-5.525 | **0.002** |
| **Testing for respiratory support** | | | |
| Comparison PIMS-all vs. KD | 2.626 | 1.132-6.092 | **0.024** |
| **Testing for inotropes / vasodilators** | | | |
| Comparison PIMS-all vs. KD | 28.554 | 2.020-36.225 | **0.004** |
| **Testing for immunoglobulins** | | | |
| Comparison PIMS-all vs. KD | 1.239 | 0.622-2.468 | 0.542 |
| **Testing for corticosteroids** | | | |
| Comparison PIMS-all vs. KD | 1.819 | 1.046-3.165 | **0.034** |
| **Testing for heparin** | | | |
| Comparison PIMS-all vs. KD | 5.554 | 1.298-23.758 | **0.021** |

**Supplemental Table 5**: Comparison of specific treatments between PIMS-all and KD patients, using logistic regression modelling.

OR, odds ratios; 95%-CI, 95%-confidence interval; Pediatric Multisystem Inflammatory Syndrome; KD, Kawasaki disease

|  | **OR** | **95%-CI** | **p-value** |
| --- | --- | --- | --- |
| **Testing for potentially irreversible cardiovascular sequelae** | | | |
| Comparison PIMS-all vs. KD | 0.833 | 0.329-2.112 | 0.700 |
| **Testing for potentially irreversible coronary artery aneurysm** | | | |
| Comparison PIMS-all vs. KD | 1.028 | 0.320-3.303 | 0.963 |

**Supplemental Table 6**: Comparison of specific outcomes between PIMS-all and KD patients, using logistic regression modelling.

OR, odds ratios; 95%-CI, 95%-confidence interval; Pediatric Multisystem Inflammatory Syndrome; KD, Kawasaki disease
